# Therapeutic Effect of Human Umbilical Cord Mesenchymal Stromal Cells Loaded With miR‐9‐5p on Hypoxic‐Ischemic Brain Damage in Neonatal Rats

**DOI:** 10.1002/brb3.71282

**Published:** 2026-02-24

**Authors:** Bin Li, Yang Hu, Lan Wang, Zhihui Dong, Can Liu, Jianwei Xu, Xuxian Wu, Hailiang Song

**Affiliations:** ^1^ Department of Pediatrics Weiyuan County People's Hospital Neijiang China; ^2^ Center for Tissue Engineering and Stem Cell Research Guizhou Medical University Guiyang Guizhou China; ^3^ Children's Medical Center Affiliated Hospital of Guizhou Medical University Guiyang Guizhou China; ^4^ Institute of Medical Science Guizhou Medical University Guiyang Guizhou China; ^5^ Department of Laboratory Medicine The First People's Hospital of Guiyang Guiyang Guizhou China; ^6^ Department of Pharmacology, School of Basic Medical Sciences Guizhou Medical University Guiyang Guizhou China; ^7^ Guizhou Guizhong Biotechnology Co., Ltd Guiyang Guizhou China; ^8^ Department of General Surgery Dongguan Dalang Hospital Dongguan Guangdong China

**Keywords:** brain protection, cytokines, human umbilical cord mesenchymal stromal cells, hypoxic‐ischemic brain damage, miR‐9‐5p

## Abstract

**Background:**

To investigate the therapeutic mechanisms of miR‐9‐5p‐overexpressing human umbilical cord mesenchymal stromal cells (hUC‐MSCs) in neonatal rat models of hypoxic‐ischemic brain damage (HIBD).

**Methods:**

Fresh neonatal umbilical cords were collected to isolate and culture human umbilical cord mesenchymal stromal cells (hUC‐MSCs). Recombinant adenovirus was used to amplify miR‐9‐5p and transduce hUC‐MSCs, generating miR‐9‐5p‐overexpressing cells. Functional assessments included: ELISA to evaluate secretory function (e.g., neurotrophic and anti‐inflammatory factors), real‐time cell analysis to measure proliferation capacity, Transwell and Dunn chamber assays to assess chemotactic migration ability. Healthy 7‐day‐old Sprague‐Dawley (SD) rats of both sexes were randomly allocated into four groups (*n* = 12/group, with 4 rats per group assigned to TTC staining, Western blot, or Morris water maze assay, respectively): Sham‐operated control group (mock surgery), Hypoxic‐ischemic brain damage (HIBD) model group, miR‐9‐5p‐hUC‐MSCs treatment group, and Adenovirus‐transduced hUC‐MSCs (Ad‐hUC‐MSCs) treatment group. The HIBD model was induced in groups 2–4. At 24 h post‐modeling, 1×10^6^ miR‐9‐5p‐hUC‐MSCs or Ad‐hUC‐MSCs were transplanted into the left lateral ventricle (injured side) of the pups. Neurological function was evaluated 48 h later using righting reflex tests for short‐term neurobehavioral assessment. Subsequently, 4 rats per group were sacrificed for 2,3,5‐triphenyltetrazolium chloride (TTC) staining to quantify cerebral infarction volume. Hippocampal tissues from another 4 rats per group were analyzed by Western blot for Beclin‐2 and Caspase‐3 protein expression. The remaining 4 rats per group underwent 28‐day Morris water maze testing for long‐term neurobehavioral evaluation.

**Results:**

Spindle‐shaped and polygonal adherent cells emerged within 3–5 days following umbilical cord tissue block inoculation, with flow cytometric analysis confirming their identity as mesenchymal stromal cells (MSCs). Compared to the Ad‐hUC‐MSCs treatment group, miR‐9‐5p enhanced the secretion of neuroreparative and anti‐inflammatory factors (e.g., NGF, BDNF, IL‐6) in hUC‐MSCs while suppressing pro‐inflammatory cytokines (e.g., IL‐1, IL‐2) (p < 0.05). Furthermore, miR‐9‐5p significantly promoted hUC‐MSCs proliferation and augmented the chemotactic migratory capacity of miR‐9‐5p‐hUC‐MSCs. At 48 h post‐transplantation in the miR‐9‐5p‐hUC‐MSCs group, the sham‐operated controls showed no detectable cerebral infarction, whereas the model group exhibited distinct pale infarct foci occupying 33.15% ± 4.38% of total brain volume (vs. controls, p < 0.05), indicating severe cerebral injury. Both miR‐9‐5p‐hUC‐MSCs and Ad‐hUC‐MSCs treatments markedly reduced infarct volumes to 14.85% ± 2.79% and 19.11% ± 4.57%, respectively, with the miR‐9‐5p‐hUC‐MSCs group demonstrating a statistically superior therapeutic effect compared to Ad‐hUC‐MSCs (p < 0.05). Transplantation of either Ad‐hUC‐MSCs or miR‐9‐5p‐hUC‐MSCs significantly improved short‐ and long‐term neurobehavioral outcomes in hypoxic‐ischemic brain damage (HIBD) rats. At 48 h post‐HIBD induction, upregulated expression of Beclin‐2 and Caspase‐3 proteins was observed in brain tissue. Notably, these elevated protein levels were attenuated following treatment with miR‐9‐5p‐hUC‐MSCs or Ad‐hUC‐MSCs.

**Conclusion:**

MiR‐9‐5p enhances the secretion of immunomodulatory factors and improves the migratory and proliferative capacities of hUC‐MSCs. Overexpression of miR‐9‐5p promotes in vivo homing of hUC‐MSCs, which mitigate cerebral injury and exert neuroprotective and reparative effects through dual mechanisms: modulating immune responses and providing neurotrophic support. Furthermore, hUC‐MSCs significantly reduce cerebral infarct volume in hypoxic‐ischemic brain damage (HIBD) rats and downregulate levels of apoptotic proteins (Beclin‐2 and Caspase‐3) in brain tissue, demonstrating potent cerebroprotective effects.

## Graphical abstract

To address the lack of effective therapies for neonatal hypoxic‐ischemic brain damage (HIBD), this study explored the therapeutic potential of miR‐9‐5p‐overexpressing human umbilical cord mesenchymal stromal cells (hUC‐MSCs). hUC‐MSCs were isolated, cultured, and transduced with miR‐9‐5p via recombinant adenovirus. Functional assays confirmed that miR‐9‐5p enhanced hUC‐MSCs' secretion of neurotrophic/anti‐inflammatory factors (NGF, BDNF, IL‐6) while suppressing pro‐inflammatory cytokines (IL‐1, IL‐2), and promoted hUC‐MSCs'. proliferation and chemotactic migration. Seventy‐two 7‐day‐old SD rats were randomized into four groups (sham, HIBD model, Ad‐hUC‐MSCs, miR‐9‐5p‐hUC‐MSCs). HIBD models were induced by left carotid artery ligation plus hypoxia, followed by intracerebroventricular transplantation of 1 × 10^6^ modified hUC‐MSCs at 24 h post‐modeling. Key results: ① miR‐9‐5p‐hUC‐MSCs significantly reduced cerebral infarct volume (14.85 ± 2.79% vs. 33.15 ± 4.38% in model group, p < 0.05) and outperformed Ad‐hUC‐MSCs (19.11 ± 4.57%, P < 0.05); ② miR‐9‐5p‐hUC‐MSCs improved short‐term (righting reflex) and long‐term (Morris water maze) neurobehavioral outcomes; ③ miR‐9‐5p‐hUC‐MSCs more effectively downregulated hippocampal apoptotic proteins (Beclin‐2, Caspase‐3) vs. Ad‐hUC‐MSCs. These findings demonstrate that miR‐9‐5p enhances hUC‐MSCs' neuroprotective efficacy via modulating immune responses, promoting neurotrophy, and inhibiting apoptosis, providing a promising therapeutic strategy for neonatal HIBD.

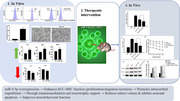

## 1 Introduction

Perinatal hypoxic‐ischemic brain damage (HIBD) in fetuses or neonates, resulting from multifactorial etiologies, represents a predominant contributor to neonatal mortality (Mo et al. 2023, Luo et al. 2023). Hypoxia‐ischemia induces developmental disturbances in immature brain tissue, subsequently manifesting as impaired consciousness, abnormal primitive reflexes, and seizures. The pathogenesis of HIBD involves inflammation, apoptosis, and oxidative stress, which collectively drive delayed neuronal death (Conway et al. 2018, Liu et al. 2021, Mwaniki et al. 2012). These pathological processes may culminate in irreversible neurodevelopmental disabilities, including intellectual impairment, cerebral palsy, behavioral disorders, and epilepsy (Qi et al. 2024, You et al. 2023, Tang et al. 2024). Notably, no clinically validated therapeutic interventions currently exist for HIBD. Emerging studies highlight mesenchymal stromal cells (MSCs)—multipotent stromal cells endowed with robust differentiation potential and immunomodulatory capabilities (Kim et al. 2022; Guenther et al. 2022)—as a novel therapeutic strategy for HIBD. While MSCs demonstrate unique potential in promoting neural regeneration and functional restoration, substantial interindividual variability in treatment efficacy has been documented, potentially attributable to heterogeneity in MSC homing capacity and paracrine functions (Wang et al. 2020; Qin et al. 2023; Jovic et al. 2022). Consequently, enhancing the migratory competence and secretory profile of MSCs to amplify their therapeutic benefits has become a pivotal research priority.

MicroRNAs (miRNAs) are endogenous non‐coding small RNAs that play pivotal roles in regulating fundamental cellular processes, including migration, intracellular trafficking, proliferation, and differentiation (Quirico et al. 2022; Kim et al. 2019; Xu et al. 2021). Our prior investigations revealed that miR‐9‐5p governs the proliferation and differentiation of neural stromal cells (NSCs), concurrently modulating neuronal migration and synaptogenesis. Notably, miR‐9‐5p is implicated in sustaining the stromalness of mesenchymal stromal cells (MSCs) and orchestrating their migratory and proliferative dynamics (Saad‐Naguib et al. 2023, Zhang et al. 2022).

In this study, human umbilical cord mesenchymal stromal cells (hUC‐MSCs) derived from Wharton's jelly of neonatal umbilical cords were utilized as seed cells. We systematically investigated miR‐9‐5p‐mediated regulation of hUC‐MSCs' secretory functions, migratory behavior, and proliferative activity. These explorations preliminarily elucidate the therapeutic role and potential molecular mechanisms of miR‐9‐5p in hUC‐MSC‐based interventions for hypoxic‐ischemic brain damage (HIBD), thereby providing empirical data and a theoretical foundation for advancing novel therapeutic strategies against HIBD.

## 2 Materials and Methods

### 2.1 Materials

7‐day‐old neonatal Sprague‐Dawley (SD) rats [Animal License No. SYXK(Gui)2023‐0001, Experimental Animal Center of Guizhou Medical University], human umbilical cords [Ethical Approval No. 2023‐16, Human Ethics Committee of Guizhou Medical University; informed consent obtained from donors], L‐DMEM culture medium, 10% fetal bovine serum (FBS), 0.25% trypsin (Thermo Fisher Scientific), serum‐free medium (Youkang Biotech), isoflurane (Lunan Pharmaceutical Group), and 2,3,5‐triphenyltetrazolium chloride (TTC; Solarbio) were utilized. qRT‐PCR primers targeting human miR‐9‐5p (miRBase Accession: [insert accession number]) and U6 reference primers were synthesized by RiboBio (Guangzhou, China). Recombinant miR‐9‐5p‐overexpressing adenovirus (Ad‐miR‐9‐5p) and empty adenovirus (Ad‐Control) previously constructed and validated in 293A cells by our group were employed. Western blot reagents were procured from Sangon Biotech (Shanghai, China). Nerve Growth Factor (NGF) ELISA kit: R&D Systems, Cat#DY256, Lot#20230512, Standard curve range: 0.156–10 ng/mL; Brain‐Derived Neurotrophic Factor (BDNF) ELISA kit: Abcam, Cat#ab212166, Lot#GR3456789‐2, Standard curve range: 15.6–1000 pg/mL; Interleukin‐1 (IL‐1) ELISA kit: Thermo Fisher Scientific, Cat#88‐7010‐22, Lot#2304158, Standard curve range: 3.9–250 pg/mL; Interleukin‐2 (IL‐2) ELISA kit: BD Biosciences, Cat#555194, Lot#7321803, Standard curve range: 7.8–500 pg/mL; Interleukin‐6 (IL‐6) ELISA kit: BioLegend, Cat#430504, Lot#B230601, Standard curve range: 1.56–100 pg/mL.

### 2.2 Methods

#### 2.2.1 Culture and Identification of hUC‐MSCs

Healthy full‐term neonatal umbilical cords with no history of infectious or hereditary diseases were collected. The Wharton's jelly was isolated from the umbilical cords and minced into tissue fragments of approximately 1 mm^3^. These fragments were seeded onto the base of culture dishes and subsequently cultured in low‐glucose DMEM (L‐DMEM) complete medium supplemented with 10% fetal bovine serum (FBS). Cultures were maintained in a humidified incubator at 37°C with 5% CO_2_. Upon reaching 40%–50% confluency, tissue fragments were removed, and cell cultures were continued until approximately 80% confluency was achieved, followed by subculturing. After 4–5 passages, cells were subjected to flow cytometric analysis for surface marker expression (CD73, CD90, CD105) to confirm their identity as human umbilical cord mesenchymal stromal cells (hUC‐MSCs).

#### 2.2.2 Preparation of hUC‐MSCs Loaded With miR‐9‐5p

At Passage 4 (P4), when hUC‐MSCs reached approximately 80% confluency, the culture medium was aspirated. Cells were washed twice with PBS, followed by the addition of 100 µL PBS containing miR‐9‐5p virus (1×10^7^ PFU/mL) to experimental groups, while control groups received an equivalent volume of adenovirus (Ad)‐containing PBS. All cultures were incubated for 1.5 h in a humidified 37°C/5% CO_2_ incubator. Viral supernatants were subsequently removed, and complete growth medium was replenished for 24–48 h of additional culture. Transduction efficiency was assessed via fluorescence microscopy by quantifying GFP‐positive cells. miR‐9‐5p expression levels in hUC‐MSCs were analyzed using quantitative reverse transcription PCR (qRT‐PCR).

#### 2.2.3 Detection of miR‐9‐5p Effects on hUC‐MSC Secretion by ELISA

The 25 cm^2^ culture flasks containing hUC‐MSCs (loaded with miR‐9‐5p and adenovirus [Ad]) at approximately 80% confluence were processed as follows: The supernatant and floating dead cells were discarded. After two washes with PBS, 3 mL of serum‐free medium was added. The supernatant was collected following 24 and 72 h of culture and analyzed for cytokines (including interleukin [IL]‐1, IL‐2, IL‐6, nerve growth factor [NGF], and brain‐derived neurotrophic factor [BDNF]) according to the ELISA kit manufacturer's protocol, and all results were normalized to the number of viable cells (per 10^5^ cells).

#### 2.2.4 Analysis of miR‐9‐5p Effects on hUC‐MSC Proliferation Using Real‐Time Cell Monitoring

hUC‐MSCs transfected with miR‐9‐5p were resuspended in complete medium at a density of 1 × 10^4^ cells/mL, with adenovirus (Ad)‐transfected cells serving as the control. A 100 µL aliquot of the cell suspension was dispensed into each well of a real‐time cell analysis electrode plate. The plate was subsequently incubated for 72 h in the xCELLigence RTCA DP System (ACEA Biosciences, San Diego, CA, USA). Cellular proliferation data were continuously recorded and normalized to the cell index (CI) at 0 h (set to 1.0); relative CI values at 24, 48, and 72 h were used to reflect proliferation rate.

#### 2.2.5 Analysis of miR‐9‐5p Effects on hUC‐MSC Chemotactic Migration Using Transwell and Dunn Chamber Assays

hUC‐MSCs transfected with miR‐9‐5p, and Ad were trypsinized using 0.25% trypsin‐EDTA, washed twice with PBS, and resuspended in serum‐free medium at a density of 2×10^5^ cells/mL. The lower chambers of Transwell plates (8‐µm pore size) received 800 µL of low‐glucose DMEM supplemented with 25 ng/mL HGF as chemoattractant. Subsequently, 100 µL cell suspension was carefully loaded into each upper chamber. Following 6‐h incubation at 37°C with 5% CO_2_, non‐migrated cells on the upper membrane surface were removed using sterile cotton‐tipped applicators. Migrated cells adherent to the lower membrane surface were fixed with 4% paraformaldehyde for 15 min, stained with 0.5% crystal violet for 30 min, and gently rinsed with PBS. Membranes were excised, mounted on glass slides, and imaged under an inverted phase‐contrast microscope (200× magnification). Cell counts from five random fields per membrane were quantified using ImageJ software (NIH).

In this study, hUC‐MSCs transfected with miR‐9‐5p and adenoviral vectors (Ad) were resuspended in serum‐free medium at a density of 1×10^4^ cells/mL and seeded onto sterile glass coverslips (Φ18 mm) for cell attachment. The Dunn chamber (ibidi) was assembled by loading complete growth medium into the inner reservoir, followed by inverted placement of the cell‐coated coverslip over the bridge assembly to establish a stable chemoattractant gradient. The chamber perimeter was sealed with high‐purity petroleum jelly to minimize evaporation and environmental perturbations. Time‐lapse imaging was performed using a live‐cell imaging system (Nikon BioStation) maintained at 37°C with 5% CO_2_, with phase‐contrast images captured every 10 min over a 4‐h period. Cell migration trajectories were analyzed using the Manual Tracking plugin in ImageJ (v1.53, NIH), and migration velocities were calculated from displacement measurements.

#### 2.2.6 Establishment of HIBD Animal Model and hUC‐MSC Transplantation Therapy

Forty‐eight healthy 7‐day‐old neonatal Sprague‐Dawley rats (mixed sex) were randomly divided into four groups (*n* = 12/group, 4 rats per endpoint, with littermates evenly distributed across groups to minimize inter‐litter variability): Sham‐operated controls, Hypoxic‐ischemic brain damage (HIBD) model group, miR‐9‐5p‐transfected hUC‐MSCs treatment group (miR‐hUC‐MSCs), and adenovirus‐transduced hUC‐MSCs treatment group (Ad‐hUC‐MSCs). A multi‐step blinding protocol was implemented: (1) Surgery (carotid ligation) and injection were performed by Researcher A (unaware of group assignments); (2) Neurobehavioral assessments were conducted by Researcher B (no access to group info); (3) TTC analysis and Western blot experiments were done by Researchers C and D (unblinded only after data compilation). Exclusion criteria: rats that died within 24 h post‐surgery (mortality rate <5%, 2/48 excluded), injection site hemorrhage, or TTC staining artifacts. The specific method is as follows: HIBD induction was performed under 3% isoflurane anesthesia via permanent ligation of the left common carotid artery. After 2‐h maternal separation, pups were exposed to hypoxia (8% O_2_/92% N_2_ at 37°C) for 60 min. At 24 h post‐modeling, transfected hUC‐MSCs (1×10^6^ cells/mL in PBS) were stereotaxically injected into the left lateral ventricle (coordinates: AP −1.0 mm, ML 1.5 mm, DV 2.5 mm relative to bregma) using a 10‐µL Hamilton syringe (5 µL/injection). The injection volume was 5 µL, delivered at a rate of 0.5 µL/min; the needle was retained for 5 min to prevent backflow, then slowly withdrawn. Injection coordinates (relative to bregma): AP −1.0 mm, ML 1.5 mm, DV 2.5 mm. Neurological assessments included acute evaluation of righting reflex latency at 48 h post‐transplantation, histopathological analysis via TTC staining of 2‐mm coronal brain sections (*n* = 4/group) for infarct volume quantification—infarct volume was calculated with edema correction: (1) Measure contralateral hemisphere volume (V_contralateral) and ipsilateral hemisphere volume (V_ipsilateral); (2) Calculate corrected ipsilateral non‐infarct volume = V_contralateral × (V_ipsilateral/V_contralateral); (3) Infarct volume (%) = [(V_contralateral—corrected ipsilateral non‐infarct volume)/V_contralateral] × 100—molecular profiling of Beclin‐2 and cleaved Caspase‐3 expression in ipsilateral hippocampal lysates by western blot (anti‐rabbit IgG‐HRP, 1:1000; *n* = 4/group), and long‐term cognitive evaluation using a 5‐day Morris Water Maze protocol in remaining animals (*n* = 4/group) at postnatal day 28.

### 2.3 Statistical Analysis

Data analysis was performed using SPSS software (v25.0, IBM). Continuous variables are presented as mean ±SEM (replaced SD). All data were tested for normality (Shapiro‐Wilk test) and homoscedasticity (Levene's test), confirming compliance with parametric test assumptions (*p* > 0.05). Between‐group comparisons were analyzed using Student's *t*‐test, while multiple group comparisons were assessed by one‐way ANOVA with post‐hoc Tukey's test. Statistical significance was defined as p < 0.05 (two‐tailed).

## 3 Results

### 3.1 Culture, Expansion, and Characterization of hUC‐MSCs

**FIGURE 1:**
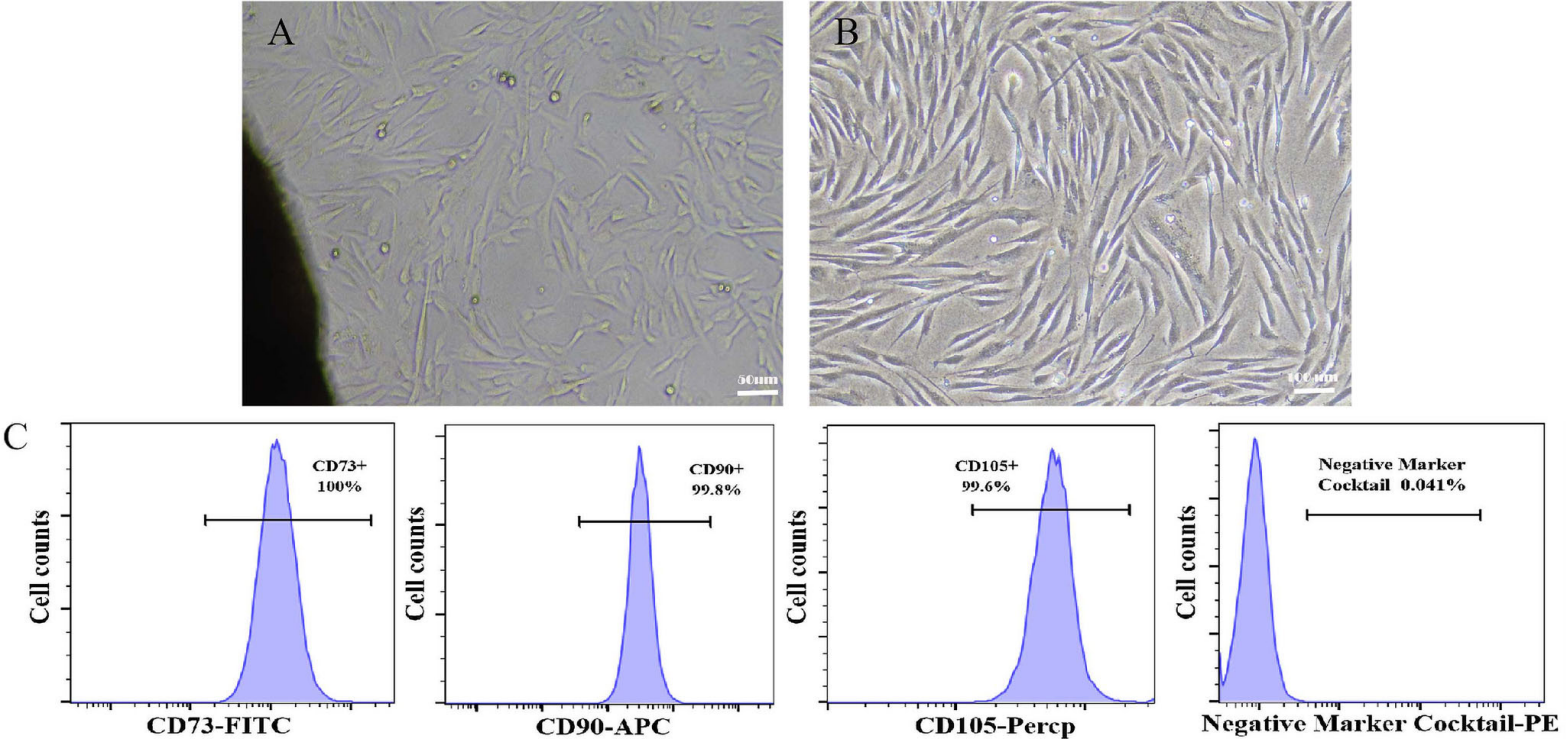
Morphological and immunophenotypic characterization of Wharton's jelly‐derived mesenchymal stem cells (hUC‐MSCs). (A)Primary cell migration from umbilical cord tissue explants (scale bar = 50 µm). (B) Morphology of passage 4 (P4) hUC‐MSCs demonstrating characteristic spindle‐shaped phenotype (scale bar = 100 µm). (C) Flow cytometric profiling showing ≥95% positivity for MSC markers (CD73/CD90/CD105) and ≤2% expression of hematopoietic markers (CD34/CD45/HLA‐DR).

Primary cells exhibiting polygonal or spindle morphology began migrating from tissue explant margins within 5–7 days post‐inoculation (Figure 1A,B). These adherent cells progressively formed confluent monolayers with characteristic whirlpool‐like arrangements. Upon reaching 70%–80% confluence within 1–2 weeks, cells were subcultured using 0.25% trypsin‐EDTA. Flow cytometric analysis of Passage 4 (P4) cells demonstrated positive expression (>95%) of MSC surface markers CD73, CD90, and CD105, while hematopoietic lineage markers (CD34/CD45/HLA‐DR) showed <2% positivity (Figure 1C), fulfilling ISCT criteria for MSCs.

### 3.2 Loading of miR‐9‐5p in hUC‐MSCs

**FIGURE 2:**
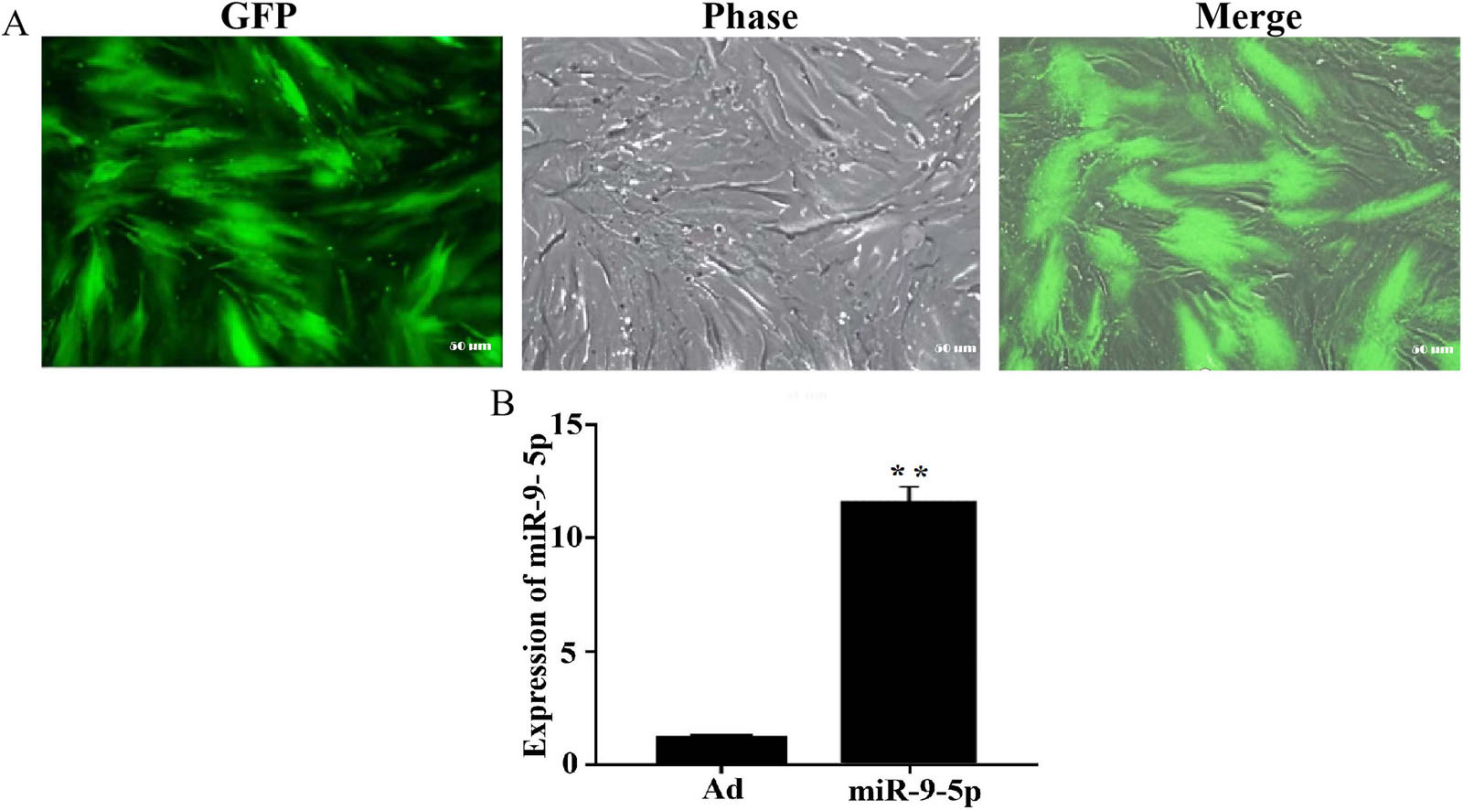
Expression of GFP and miR‐9‐5p in hUC‐MSCs 48 h post‐transfection. (A) GFP expression in hUC‐MSCs 48 h post‐transfection with miR‐9‐5p (scale bar = 50 µm). (B) miR‐9‐5p expression levels in hUC‐MSCs (*n* = 3 independent cell culture experiments; statistical test: Student's *t*‐test; exact *p* value: *p* = 0.008 vs. Ad‐hUC‐MSCs). ** Denotes statistical significance compared to the control group (p < 0.01).

After 24 h of adenoviral infection (carrying miR‐9‐5p and Ad), green fluorescence signals were observed in hUC‐MSCs under fluorescence microscopy, confirming successful cellular uptake and expression of miR‐9‐5p and Ad. By 48 h post‐infection, over 70% of the cells exhibited green fluorescence (Figure 2A). qRT‐PCR analysis further demonstrated significantly higher miR‐9‐5p expression in the miR‐9‐5p‐hUC‐MSCs group compared to the Ad‐hUC‐MSCs group, supporting their suitability for subsequent experiments (p < 0.01, Figure 2B).

### 3.3 Effects of miR‐9‐5p on hUC‐MSC Secretion

**FIGURE 3:**
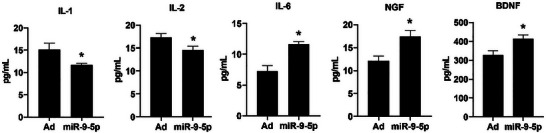
Cytokine content in supernatant of serum‐free culture. Cytokine concentrations (normalized to viable cells) in 24 and 72 h serum‐free culture supernatant (*n* = 3 independent cell culture experiments; ^*^ *p* < 0.05 vs. Ad‐hUC‐MSCs [NGF: *p* = 0.012, BDNF: *p* = 0.009, IL‐6: *p* = 0.015, IL‐1: *p* = 0.023, IL‐2: *p *= 0.018 at 72 h]).

Supernatant samples from the miR‐9‐5p‐hUC‐MSCs group and Ad‐hUC‐MSCs group were collected after 72 h of serum‐free culture. The concentrations of nerve growth factor (NGF), brain‐derived neurotrophic factor (BDNF), interleukin‐1 (IL‐1), interleukin‐2 (IL‐2), and interleukin‐6 (IL‐6) were measured using ELISA. Results demonstrated that compared with the Ad‐hUC‐MSCs group, the miR‐9‐5p‐hUC‐MSCs group showed significantly increased secretion of NGF, BDNF, and IL‐6, along with significantly decreased secretion of IL‐1 and IL‐2, with all differences being statistically significant (p < 0.05, Figure 3).

### 3.4 Effects of miR‐9‐5p on the Proliferative Capacity of hUC‐MSCs

**FIGURE 4:**
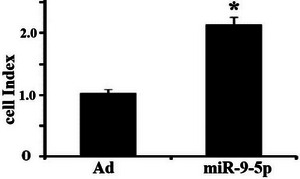
Effects of miR‐9‐5p on hUC‐MSCs proliferation. Relative cell index (normalized to 0 h) of hUC‐MSCs over 72 h (*n* = 3 independent cell culture experiments; *p* = 0.021 at 48 h, *p* = 0.015 at 72 h vs. Ad‐hUC‐MSCs).

Proliferation assays using a real‐time cell analysis system revealed that miR‐9‐5p‐hUC‐MSCs demonstrated significantly enhanced proliferative capacity compared to Ad‐hUC‐MSCs during the 72‐h observation period (Figure 4).

### 3.5 Effect of miR‐9‐5p Loading on hUC‐MSCs Migration

**FIGURE 5:**
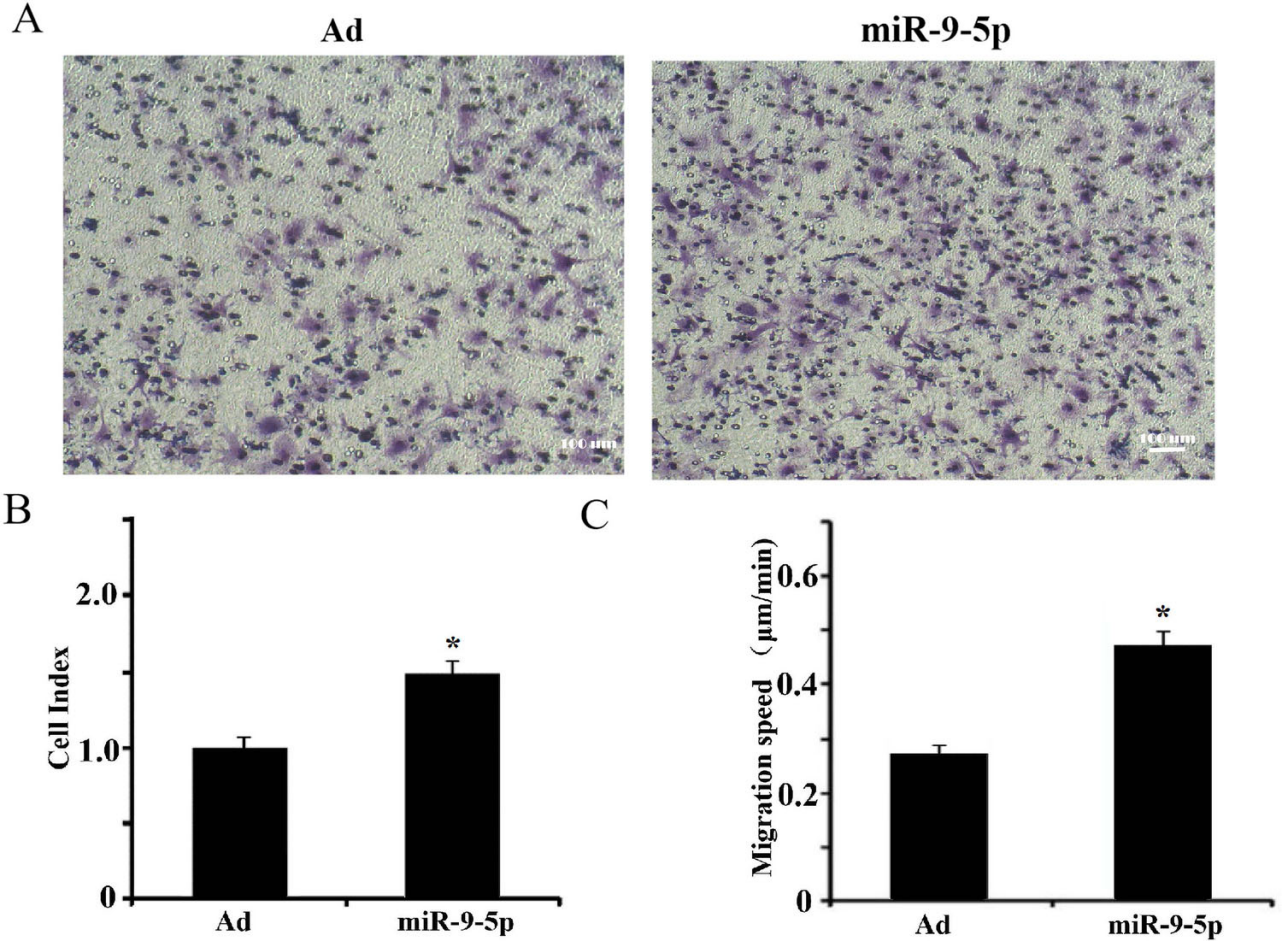
Effects of miR‐9‐5p on hUC‐MSC chemotactic migration. (A) Phase‐contrast microscopy of transmigrated hUC‐MSCs (Transwell assay, scale bar = 100 µm). (B) Transwell migration efficiency (number of migrated cells per field). (C) hUC‐MSCs migration rate (µm/h) (Dunn‐chamber assay) (*n* = 3 independent cell culture experiments; B: *p* = 0.021, C: *p* = 0.018 vs. Ad‐hUC‐MSCs).

In Transwell chemotaxis assays, miR‐9‐5p‐hUC‐MSCs showed enhanced directional migration after 6 h of incubation (Figure 5A,B). Dunn‐chamber assays further quantified hourly migratory velocity, revealing significantly higher migration rates in miR‐9‐5p‐hUC‐MSCs compared to Ad‐hUC‐MSCs (*p* < 0.05; Figure 5C).

### 3.6 Colonization of hUC‐MSCs in the Brain and TTC Staining Results

**FIGURE 6:**
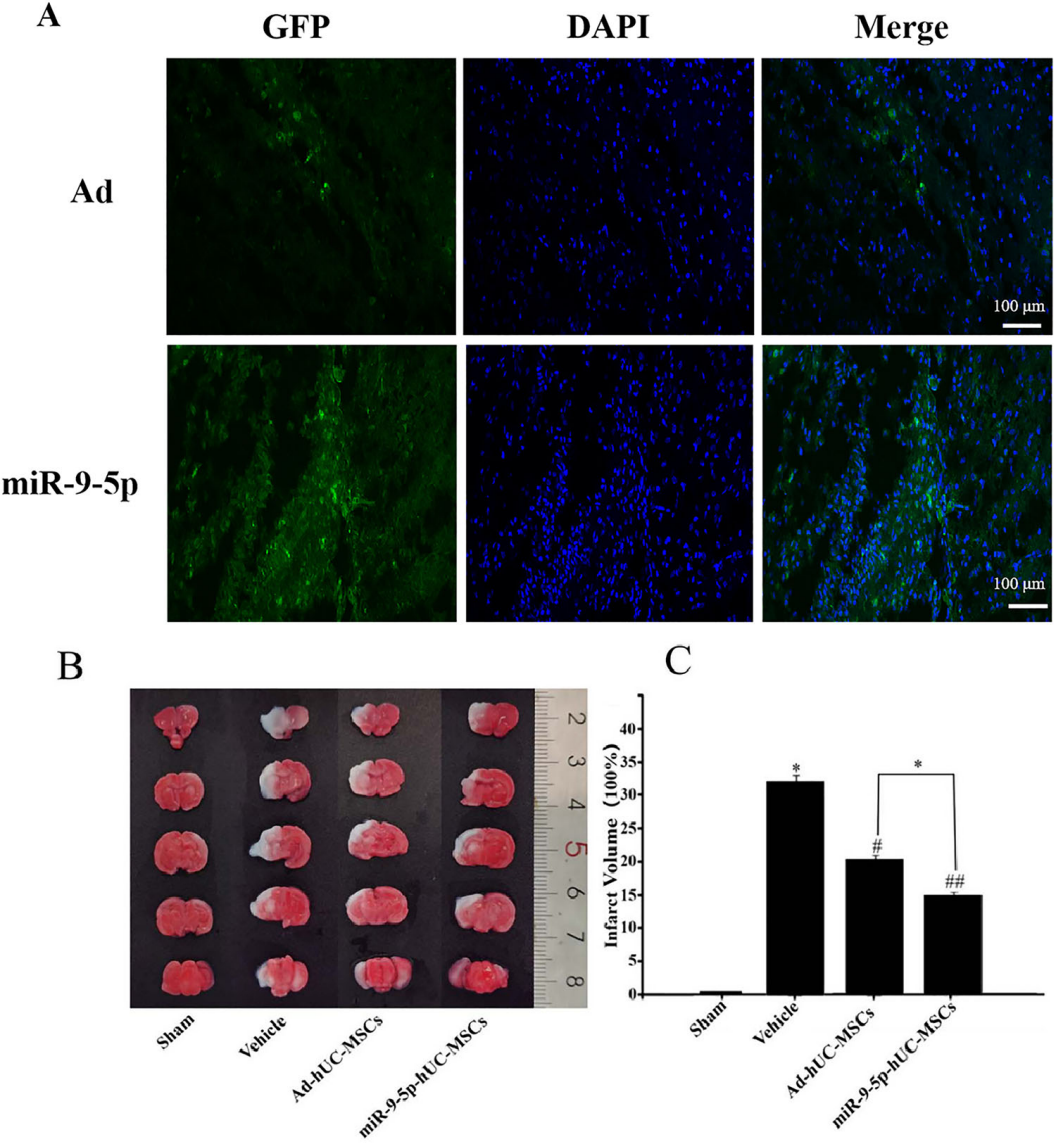
Colonization of hUC‐MSCs in the brain and TTC staining of rat brain in each group. (A) Colonization of hUC‐MSCs in the brains of HIBD rats (×50; green: GFP‐positive hUC‐MSCs, blue: DAPI‐stained nuclei; arrows indicate hUC‐MSCs; scale bar = 20 µm). (B) TTC staining. (C) Proportion of infarct area (*n* = 4 rats per group; compared with the control group, ^*^ *p* < 0.05; compared with the model group, ^#^ *p* < 0.05, ^##^ *p *< 0.01; miR‐9‐5p‐hUC‐MSCs vs. Ad‐hUC‐MSCs: *p* = 0.032).

Forty‐eight hours after transplantation in the miR‐9‐5p‐hUC‐MSCs and Ad‐hUC‐MSCs groups, fluorescence microscopy revealed numerous green‐fluorescent cells distributed around the left lateral ventricle injection site in rats, indicating successful migration and colonization of hUC‐MSCs in the brain tissue (Figure 6A). TTC staining results showed that the model group exhibited distinct cerebral infarction foci with an infarct volume ratio of 33.15% ± 4.38%. Both treatment groups showed significant reduction in infarct volumes: 14.85% ± 2.79% in the miR‐9‐5p‐hUC‐MSCs group and 19.11% ± 4.57% in the Ad‐hUC‐MSCs group, with a statistically significant difference between the two treatment groups (*p *< 0.05, Figure 6B,C).

### 3.7 Effects of hUC‐MSC Transplantation on Neurobehavioral Function in HIBD Rats

**FIGURE 7:**
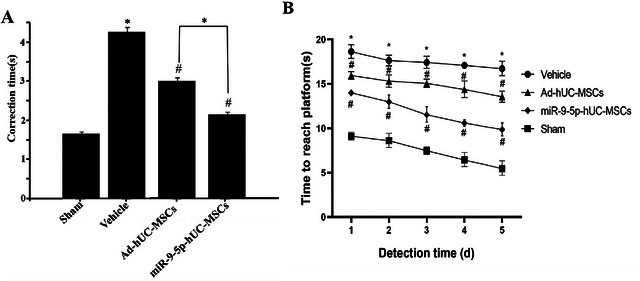
Neurobehavioral assessment results of experimental groups. (A) Righting reflex experiment (48 h post‐transplantation); (B) Water maze experiment (postnatal day 28); (*n* = 4 rats per group; compared with the normal control (sham operation) group, ^*^ *p* < 0.05; compared with the model group, ^#^ *p* < 0.05, ^##^ *p *< 0.01; miR‐9‐5p‐hUC‐MSCs vs. Ad‐hUC‐MSCs: *p* = 0.028).

Twenty‐four hours post‐HIBD modeling, righting reflex tests revealed significantly prolonged righting time in model group rats compared to sham controls (*p* < 0.05). At 48 h post‐transplantation, miR‐9‐5p‐hUC‐MSC recipients showed more pronounced functional recovery than Ad‐hUC‐MSC‐treated (Figure 7A) rats. In 28‐day Morris water maze assessments, HIBD rats demonstrated significant cognitive impairment (longer platform latency vs. controls, *p* < 0.01). Both transplantation groups showed reduced escape latencies, with miR‐9‐5p‐hUC‐MSCs exhibiting superior improvement (Figure 7B).

### 3.8 Effects of hUC‐MSCs Transplantation on the Expression of Beclin‐2 and Caspase‐3 Proteins in the Hippocampal Tissue of HIBD Rats

**FIGURE 8:**
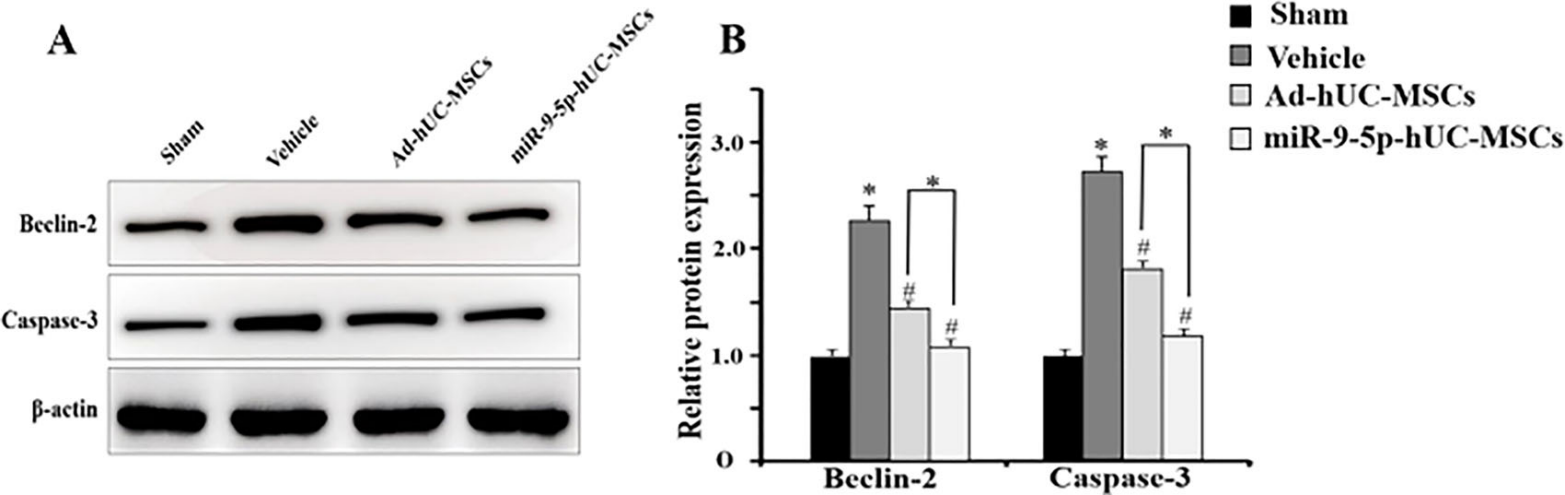
Expression of Beclin‐2 and Caspase‐3 proteins (*n* = 4 rats per group; compared with the normal control sham group, ^*^ *p* < 0.05; compared with the model group, ^#^ *p* < 0.05; miR‐9‐5p‐hUC‐MSCs vs. Ad‐hUC‐MSCs: Beclin‐2: *p* = 0.031, Caspase‐3: *p* = 0.027).

At 48 h after HIBD modeling, the expression of Beclin‐2 and Caspase‐3 proteins in the brain tissue was significantly upregulated (Figure 8A). Forty‐eight hours after the transplantation of miR‐9‐5p‐hUC‐MSCs and Ad‐hUC‐MSCs, the expression of Beclin‐2 and caspase‐3 proteins was significantly lower than that in the model group (#*p* < 0.05). Compared with the Ad‐hUC‐MSCs group, the expression of Beclin‐2 and caspase‐3 proteins in the miR‐9‐5p‐hUC‐MSCs group was significantly decreased (^*^ *p* < 0.05).

## 4 Discussion

This study aimed to investigate the role of miR‐9‐5p in human umbilical cord mesenchymal stromal cells (hUC‐MSCs) for treating neonatal hypoxic‐ischemic brain damage (HIBD) and its underlying mechanisms. First, we successfully isolated and cultured hUC‐MSCs from neonatal umbilical cords and constructed miR‐9‐5p using recombinant adenovirus (AD) technology, ensuring effective miR‐9‐5p delivery by hUC‐MSCs. Functional studies revealed that compared to empty‐vector‐transfected Ad‐hUC‐MSCs, miR‐9‐5p‐loaded hUC‐MSCs significantly enhanced secretion of key cytokines, including interleukin‐6 (IL‐6), nerve growth factor (NGF), and brain‐derived neurotrophic factor (BDNF), which play crucial roles in anti‐inflammation, tissue repair, and angiogenesis (Wei et al. 2023, Kerkis et al. 2024, Liu et al. 2022). This miR‐9‐5p‐mediated regulation of hUC‐MSCs' secretory function may help ameliorate the post‐HIBD inflammatory microenvironment and accelerate neural repair. Additionally, miR‐9‐5p promoted hUC‐MSCs proliferation (He et al. 2024, Montalbán‐Hernández et al. 2022). As cell proliferation is fundamental for tissue regeneration, this effect could enhance therapeutic outcomes. The study also demonstrated that miR‐9‐5p‐hUC‐MSCs exhibited significantly improved chemotactic migration capacity, with both migration efficiency and velocity markedly increased, suggesting miR‐9‐5p's critical role in regulating hUC‐MSCs motility. Existing evidence indicates that tissue repair efficacy correlates with homing cell numbers, which largely depend on cellular migration capacity. Neurotrophic factors like NGF and BDNF not only promote hUC‐MSCs migration to lesion sites but also facilitate their differentiation (Ko et al. 2018, Hu et al. 2019).

In animal experiments, transplantation of miR‐9‐5p‐modified hUC‐MSCs into the ipsilateral ventricle of HIBD model rats resulted in significantly smaller cerebral infarction volumes compared to both the model group and Ad‐hUC‐MSCs group. Neurobehavioral assessments demonstrated that both Ad‐hUC‐MSCs and miR‐9‐5p‐hUC‐MSCs transplantation improved neurological function in HIBD rats during both short‐ and long‐term observation periods, with miR‐9‐5p‐hUC‐MSCs exhibiting superior therapeutic effects. These findings indicate that miR‐9‐5p overexpression significantly enhances hUC‐MSCs' capacity for repairing hypoxic‐ischemic neural damage, providing neuroprotection that not only reduces infarction volume and mitigates brain tissue injury but also leads to marked improvement in neurobehavioral outcomes. Beclin‐2 and Caspase‐3 protein expression was significantly upregulated at 48 h post‐HIBD modeling but decreased following hUC‐MSCs treatment, suggesting hUC‐MSCs modulate these apoptosis‐related proteins during brain repair in neonatal HIBD rats, thereby inhibiting neuronal apoptosis. Studies confirm that Beclin‐2 regulates both apoptosis and autophagy through interactions with Class III PI3K complex and Bcl‐2 (Xu et al. 2019, He et al. 2013, Galluzzi and Kroemer 2013). As the key executioner in caspase cascade, Caspase‐3 participates in neuronal apoptosis and can be activated directly or indirectly by various factors, including cytochrome C, TNF‐α, ROS, IL‐1, and IL‐2 (Li et al. 2023, Sokolova et al. 2022). Notably, miR‐9‐5p‐hUC‐MSCs showed more pronounced reduction in these apoptosis‐related proteins compared to Ad‐hUC‐MSCs, suggesting miR‐9‐5p may synergistically enhance hUC‐MSCs' anti‐apoptotic effects, reducing infarction volume and hypoxic‐ischemic damage while promoting functional recovery in HIBD rats. The neuroprotective effects of hUC‐MSCs—mediated through secretion, differentiation, and immunomodulation—are well established (Sun et al. 2023, Xu et al. 2024). Our study further confirms hUC‐MSCs' brain‐protective effects in HIBD rats, with miR‐9‐5p overexpression providing additional therapeutic optimization.

Our study focused on 24 h post‐HIBD transplantation (a critical window for secondary injury mitigation, which peaks at 24–72 h post‐insult [Liu et al., 2021, CNS Neurosci Ther]) and 48 h/28 d endpoints. The reduced infarct volume in miR‐9‐5p‐hUC‐MSCs‐treated rats (48 h post‐transplantation) primarily reflects mitigation of secondary injury (e.g., inflammation, apoptosis). Future studies will include 24 h baseline TTC staining and longitudinal MRI to further validate this mechanism.

In summary, this study elucidates the pivotal role of miR‐9‐5p in human umbilical cord mesenchymal stromal cell (hUC‐MSC)‐based therapy for hypoxic‐ischemic brain injury (HIBD). Our findings demonstrate that miR‐9‐5p significantly enhances three critical therapeutic properties of hUC‐MSCs: (1) migratory capacity, (2) proliferative potential, and (3) secretory function, collectively improving their neurorestorative efficacy in HIBD model rats. These modifications led to measurable neuroprotection, including reduced brain tissue damage and significantly improved neurobehavioral outcomes. Mechanistically, we identified a synergistic interaction between hUC‐MSCs and miR‐9‐5p that primarily exerts neuroprotective effects through suppression of apoptotic pathways. This work establishes important experimental evidence for developing innovative therapies against HIBD and related neurological disorders.

## Author Contributions

Bin Li and Yang Hu conceived and designed the study and drafted the manuscript. Lan Wang and Zhihui Dong performed experiments and data analyses. Can Liu, Jianwei Xu, Xuxian Wu, and Hailiang Song provided critical comments, funding acquisition, and revisions on the manuscript. All authors read and approved the final manuscript.

## Conflicts of Interest

The authors declare no conflicts of interest.

## Data Availability

The data that support the findings of this study are available from the corresponding author upon reasonable request.
